# Parental perspectives on brain health education for primary school-aged children: a cross-sectional study

**DOI:** 10.3389/fpubh.2026.1829475

**Published:** 2026-07-06

**Authors:** Lily A. Montague, Michelle Catanzaro, Joyce Siette

**Affiliations:** 1 The MARCS Institute for Brain, Behavior and Development, Western Sydney University, Westmead, NSW, Australia; 2 School of Arts, Western Sydney University, Ryde, NSW, Australia

**Keywords:** brain health, children, dementia risk, parental acceptance, primary school, public health

## Abstract

**Background:**

Up to 45% of dementia risk is considered modifiable across the life course. Research demonstrates that exposure to education in childhood can increase positive brain health outcomes in later life. While mid- and late-life educational interventions have been extensively studied, there is limited empirical evidence examining the appropriateness of education programs, including prevention approaches initiated in childhood, particularly those delivered through schools and supported by families. Understanding parental perspectives provides insights into how early-life brain health programs can be implemented. This study explored parental views and willingness to engage in primary school-based brain health educational programs and identified perceived barriers, facilitators, and preferred program components.

**Methods:**

Urban and suburban parents (*N* = 444, Mean age = 40.6, SD = 5.5, 95% female) of primary school-aged children participated in an online survey on program appropriateness, usefulness, barriers and preferred content, delivery modalities, and features. Descriptive statistics and a multiple linear regression analysis was used to identify sociodemographic factors associated with program acceptance. Content analysis was used to analyze open-ended responses.

**Results:**

Most participants endorsed brain health education for their primary school-aged children (83%), with all perceiving a prospective program as useful for their children (100%) and almost all for themselves as parents (99.5%). Higher program endorsement was associated with fewer perceived barriers and greater perceived usefulness for child development and parenting practices (*p* < 0.001). Three main themes were identified: Family and program barriers (e.g., cost, non-engaging materials), family and program facilitators (e.g., valuing healthy ageing, use of evidence-based information) and the need for tailored programs with integrated involvement (e.g., home and school).

**Conclusion:**

Parents strongly valued education on brain-healthy behaviors and described childhood as a promising window for dementia risk reduction for their children. Future programs should be comprehensive, engaging and tailored, with explicit strategies to connect school-based learning into the home. These findings contribute to the growing life-course risk reduction agenda.

## 1 Introduction

Despite growing evidence that dementia risk can be modified, Australia continues to experience a disproportionate burden of dementia relative to global health trends (1, 2). Robust evidence indicates that higher levels of education before adulthood have preventative benefits and are recognized as the third largest modifiable risk factor for dementia (2). As such, emerging research is increasingly shifting focus toward targeting early-life health behaviors and fostering protective habits as an avenue to prevent risk of later cognitive decline (3–5).

In Australia, several national and state-level school-based health promotion initiatives exist to address a range of health-related concerns among children (6–9). While commendable, these initiatives typically operate independently, with each focusing on a narrow facet of health promotion, such as physical activity, healthy eating, or wellbeing. An overarching framework that integrates health-related behaviors within the context of lifelong brain health is unfortunately absent, although recent commentaries have begun to address this gap (10, 11). Brain health educational programs offer a promising avenue by providing a cohesive, lifespan-oriented approach to health promotion and habit formation. Recent studies are beginning to explore the feasibility and acceptability of introducing such programs during early childhood (3–5). Early childhood, the period from conception to 8 years of age (12) holds a significant influence on a child’s life course and developmental trajectory (12). Investigations within this developmental period have been positively received by parents, who largely agree on the value, relevance and necessity of such knowledge and practices for their children (3, 4). Despite a myriad of potential barriers such as time, cost and access, parents acknowledged that programs educating children on brain-healthy behaviors are missing elements in their education. With such programs being well received among early childhood parents, it is important to explore whether these attitudes exist among primary school parents to further strengthen the brain health and dementia prevention agenda.

It is known that parents are uniquely positioned to influence both their own and their children’s health outcomes (3). For brain health educational programs, parents’ intended engagement may depend on factors such as past exposure to dementia, level of understanding or awareness of dementia, perceived barriers and usefulness of a program (3, 4). In addition to these factors, parental adoption of health promotion content for children is frequently compromised by a range of structural, psychosocial and contextual barriers (13, 14).

Research in early childhood settings suggests that parents often encounter challenges when supporting their children’s healthy behaviors. These barriers include time constraints, competing priorities, financial limitations, lack of behavioral control, and children’s resistance to change (15, 36). Additionally, parents with limited awareness or knowledge of health-promoting practices and available programs are less likely to engage with them (4, 16). Conversely, when parents are equipped with accessible, trustworthy information, engaging resources, and interventions that align with their values, lived realities, and offer opportunities for involvement, their motivation for adopting and sustaining health-promoting behaviors for their children increases (4). However, with the focus of these studies being on early childhood, it remains unclear whether these barriers and facilitators are relevant to parents of primary school-aged children. Therefore, it is essential to consider parents’ perceived barriers and facilitators when designing equitable and sustainable brain health programs that extend beyond the classroom for this cohort to ensure parental involvement is optimized.

Some studies have piloted and examined intergenerational programs designed to educate primary school-aged children about dementia. These initiatives have been effective in increasing children’s understanding of what dementia is (17) and fostering greater empathy toward people living with or affected by it (18). However, most efforts have prioritized raising awareness of dementia itself and promoting compassionate attitudes, with comparatively little attention given to dementia risk reduction, or practical ways children can support their own brain health. Given this, there remains a gap in evidence regarding educational programs tailored to primary school-aged children on brain-healthy practices. As such, it is essential to first explore the key program elements such as topics, delivery settings, and approaches before designing and developing such an initiative.

The present study thus aimed to (i) explore factors that influence parents’ intended uptake of brain health programs for their primary school-aged children; (ii) identify parents’ perceived barriers and facilitators to the adoption and implementation of a brain health program; and (iii) understand parents’ preferences for the location, content and modality of a potential brain health program.

## 2 Methods

### 2.1 Study design

The study utilized a 15-minute, online, anonymous, cross-sectional survey containing both open-and-closed-ended questions. The study was approved by the Western Sydney University Human Research Ethics Committee (reference H15440).

### 2.2 Participants

Parents with at least one child that attended primary school were recruited across Australia. Alongside word of mouth, electronic flyers were distributed to before and after school care centres, private primary schools, child psychology clinics and parent groups on social media. E-newsletters were circulated via the research team’s networks, including Western Sydney’s E-update, the Young and Resilient Research Centre, and the MARCS Institute’s BabyLab.

Eligible participants were parents aged over 18 years, could communicate in English, and were able to provide informed consent. To encourage survey completion, both monetary and non-monetary incentives were offered. Two $100 e-gift vouchers were randomly provided to participants who completed the survey at the conclusion of the study. Non-monetary incentives included two brain health educational activity booklets for their child(ren) and a parent information sheet on brain health and the research team’s existing work, designed by the team upon survey completion.

### 2.3 Materials and procedure

The online survey was available from May 2025 to August 2025 and was created using the Qualtrics platform. A reCAPTCHA authentication was required to commence the survey eliminating risk of bots and malicious operators. The survey included 47 adaptive closed-ended and 10 adaptive open-ended questions, mostly presented one per page or grouped when related. Closed-ended questions required a mandatory response, typically with multiple options, while open-ended questions could be skipped to reduce attrition. Participants could review their answers using a back button. The survey consisted of seven blocks designed to capture parents’ perspectives on brain health education and was informed by the research team’s previous survey for preschool-aged children (4). Items were adapted from this prior survey ensuring content validity, with an iterative review of the modifications to reflect findings from earlier work, and to ensure relevance for Australian primary school settings (4). As the study was designed to be descriptive and exploratory, the measures were not designed as formal psychometric instruments, and no formal validation procedures were undertaken. Each survey block is described below, and a copy of the survey is available in the Supplementary material.

*Demographics:* Questions included participants’ age, gender, place of birth, postcode, highest education level, employment and family structure. Questions also asked about whether participants spent time meaningfully with their child(ren), their child(ren)‘s current lifestyle habits (e.g., sleep hygiene, physical activity levels) and current level of brain health education. Other questions assessed participants’ exposure to someone with a brain-related disease and whether their child is neurodiverse.

*Willingness to participate and usefulness of brain health programs:* Assessed participants intended uptake and usefulness of a brain health program for themselves and their child(ren). Responses were measured using a 5-point Likert scale (‘not useful at all’ to ‘extremely useful’). Participants who responded ‘not useful at all’ were prompted with a follow-up open-ended question to explain their selection. Similarly, intended uptake of a program was assessed using a 5-point Likert scale (‘extremely unlikely’ to ‘extremely likely’).

*Modalities, location and setting preferences:* Asked questions related to program delivery modalities (e.g., storybook, film, electronic application) and locations (e.g., home, primary school, holiday program) most preferred by participants. Participants were asked to rank modalities in order of preference but not location. Three open-ended questions requested participants’ insights and suggestions for each area (e.g., modality, location, program setting).

*Program content:* Explored participants’ opinions on various topics and features of a program. A 5-point Likert scale (‘strongly disagree’ to ‘strongly agree’) was used to explore topics (e.g., social engagement, sleep hygiene, stress management). The same scale was used for exploration of features of a program (e.g., user-friendly materials and affordability). Two open-ended questions provided participants with the opportunity to offer further suggestions on topics or features.

*Barriers and facilitators:* Statements describing 10 potential barriers were displayed, for example, *‘I do not have time to implement a program at home’,* with participants asked to rate their agreeableness using a 5-point Likert scale (‘strongly agree’ to ‘strongly disagree’). Open-ended questions followed exploring additional barriers identified by participants for both short-term and long-term implementation. For example, *‘What barriers, if any, do you think might make it difficult to implement these changes at home with your child(ren)?’.* To explore facilitators, participants were asked about specific motivators to make healthy lifestyle changes, as well as features of a program that would increase implementation, with an option to elaborate using an open-ended question.

### 2.4 Analytical design

#### 2.4.1 Quantitative analysis

Descriptive statistics were used to describe the dataset (e.g., mean, standard deviation, frequency, percentage of responses) for demographics (e.g., gender, age, socioeconomic status), intended uptake, usefulness, preferred modality, content, delivery preferences, barriers and facilitators. Participants’ socioeconomic status was calculated using the Index of Relative Socio-economic Advantage and Disadvantage (IRSAD) based on postcode data, grouped in quantiles (19) as per previous surveys (4, 20, 21). A multiple regression analysis was employed to investigate factors influencing intended uptake of brain health programs, with total combined barriers (up to 10), parent gender, parent age, socioeconomic status, education, average child age, personal motivators, perceived usefulness for child and parent as predictors. Predictors were selected based on conceptual relevance, and aligned with socio-ecological frameworks, incorporating individual, interpersonal and contextual factors (22, 23). The sample size was adequate for multiple regression, with the ratio of cases to predictors exceeding recommended established guidelines (*N* ≥ 50 + 8 *m*) (24). With nine predictors (*m* = 9), a minimum sample size of 122 was required (24), indicating sufficient power to detect meaningful effects. The significance level was set at 0.05 (𝛂) and covariates of parent age and gender were controlled for given their established association with health-related decision making. Prior to analysis, multiple regression assumptions of independence of observations, homoscedasticity, linearity, normality of residuals, and absence of multicollinearity and singularity were checked and adequately met. Six cases were considered outliers and excluded from further analysis, with *z*-scores deviating outside the ± 3.29 range (24). An additional four cases were excluded due to information on age and gender not provided. Therefore, bootstrapping was conducted to obtain robust confidence intervals in the final analysis (25). Descriptive statistics and multiple regression analysis were performed using SPSS (39).

#### 2.4.2 Qualitative analysis

Qualitative content analysis was employed to analyze the open-ended responses using an inductive approach. Analysis began with repeated reading of participants’ responses to become familiar with the data. This was followed by open-ended coding guided by the content of the responses (26). Initial categories were identified by grouping codes with similar meaning and organized into a hierarchical structure comprising first-order categories (e.g., for barriers: parent, child, program, and ambiguous barriers) and second-order categories (e.g., time/routine, interest, child behavior, cost, knowledge, and motivation). In line with established approaches and frameworks, categories were conceptualized as domain summaries derived from participants’ responses and constituted the primary output of the analysis.

Categories and themes were reviewed, refined, and defined through an iterative and collaborative process. To ensure coding reliability, the identification of codes and categories was conducted with a focus on agreement between multiple coders (LM, JS). Inter-rater reliability was assessed using Cohen’s kappa (27). The agreement among the two coders was almost perfect (*κ* = 0.87), with an observed agreement of 94.4%. Responses could be assigned to multiple categories where appropriate to capture their full meaning (e.g., *“Time and if they [child] resist or do not want to…”* was assigned to ‘interest’, ‘difficult behavior’ and ‘time’). Neutral or ambiguous responses that did not adequately address the survey question (e.g., responses that were too vague or too short to interpret) were excluded from further analysis (e.g., *“good enthusiastic mum,” “not much”*) following agreeance between coders on the neutral responses. Using a mixed-methods approach, qualitative data were transformed into quantitative data via frequency counts to determine the prevalence of each category, consistent with qualitative content analysis and previous research analysing parent survey data (28, 37). Data management and analyses were conducted using a purpose-designed Microsoft Excel (Version 16.51) spreadsheet.

## 3 Results

### 3.1 Sample characteristics

A total of 626 participants accessed the survey, with 444 completing it, resulting in a 70.9% completion rate (*M*_age_ = 40.6, SD = 5.5). Participant characteristics are presented in Table 1. A majority of participants were aged between 35 and 44 years (68%), women (95%) and living in a high socioeconomic postcode (34%). Some had completed an undergraduate/graduate-level degree as their highest level of education (67.8%) and were born in an English-speaking country (84%). Most respondents were working (89.6%), with a majority married or in de facto relationships (90.6%). Over half (57.9%) reported having a relative or someone they know suffer from a brain disease and over a quarter (31.5%) reported their child(ren) identify as neurodiverse.

**Table 1:** Summary of participant sample characteristics (*N* = 444).

Variable	*N* (%)	M (SD)
Age (years)		40.6 (5.5)
25–34	49 (11)	
35–44	302 (68)	
45–54	87 (19.6)	
55–74	5 (1.1)	
Prefer not to say	1 (0.2)	
Gender		
Male	20 (4.5)	
Female	422 (95)	
Prefer not to say	2 (0.5)	
Country of birth		
English-speaking country	373 (84)	
Non-English-speaking country	71 (16)	
Socioeconomic status		
1 (lowest)	50 (11.3)	
2	73 (16.4)	
3	101 (22.7)	
4	67 (15.1)	
5 (highest)	151 (34)	
Highest level of education		
Non-graduate degree	138 (31.1)	
Undergraduate/graduate degree	301 (67.8)	
Prefer not to say	5 (1.1)	
Working status		
Working	394 (89.6)	
Non-working	39 (8.8)	
Prefer not to say	7 (1.6)	
Family structure		
Married/De facto	402 (90.6)	
Single/other	43 (9.4)	
Average age of children		7.9 (2.76)
Children identified as neurodiverse		
Yes	140 (31.5)	
No	294 (66.2)	
Prefer not to say	10 (2.3)	
Know someone with brain disease		
Yes	257 (57.9)	
No	185 (41.7)	
Prefer not to say	2 (0.5)	

A majority (83.1%) of participants were willing for their children to participate in a prospective brain health program, although all believed a program would be useful for their child(ren) to some extent (100%) (Table 2). Almost all believed a program would be useful for themselves as parents (99.5%), with over half of the participants’ child(ren) already exposed to brain health information (64.2%). Almost all participants favoured primary school as the location for a program (91.9%). Of this number, almost half favoured a program solely at school (44.8%), with the other half preferring a program both at school and home (46.6%) (Table 2).

**Table 2:** Summary of existing exposure to brain health education, parental intended uptake, parental usefulness, child usefulness, motivation, and location preferences (*N* = 444).

Variable	*N* (%)
Child exposed to brain health information	
Yes	285 (64.2)
No	159 (41.7)
Prefer not to say	2 (0.5)
Intended uptake of brain health program	
Extremely unlikely	0 (0)
Unlikely	7 (1.6)
Neutral	68 (15.3)
Likely	241 (54.3)
Extremely likely	128 (28.8)
Perceived usefulness for child	
Not useful at all	0 (0)
Slightly useful	23 (5.2)
Moderately useful	90 (20.3)
Very useful	210 (47.3)
Extremely useful	121 (27.3)
Perceived usefulness for parent	
Not useful at all	2 (0.5)
Slightly useful	21 (4.7)
Moderately useful	78 (17.6)
Very useful	212 (47.7)
Extremely useful	131 (29.5)
Program location preferences	
Primary school	408 (91.9)
Home	227 (51.1)
Before and after school care	91 (20.5)
Holiday program	106 (23.9)
Other	23 (5.2)
Existing motivators to program uptake	
Yes	315 (70.9)
No	129 (29.1)

When identifying children’s most common existing lifestyle behaviors, 87.6% reported their child(ren) always or often have good socialization with peers, 83% reported good sleep hygiene, and 82.5% reported appropriate physical activity levels (Table 3). Participants preferred the program to be delivered via storybooks (17.3%), followed by a short film (14.2%) and an electronic application (12.6%) (Table 4). They also reported that a program should have user-friendly materials (97.5%), be easily accessible (96.2%), and affordable (94.4%) with parental involvement (89.4%) (Table 5). Participants reported high interest in all presented topics, ranging from 94 to 96.4% agreement (Table 6). The most prevalent perceived barrier was lack of accessibility (94.1%), parental lack of knowledge about a program and its outcomes (81.5%), and program cost (55.6%) (Table 5).

**Table 3:** Summary of primary school children’s existing habits (*N* = 444) [*N* (%)].

Variable	Always	Often	Sometimes	Rarely	Never
Sleep hygiene	203 (45.7)	168 (37.8)	59 (13.3)	10 (2.3)	3 (0.7)
Physical activity	193 (43.5)	173 (39)	68 (15.3)	10 (2.3)	0 (0)
Healthy eating	90 (20.3)	227 (51.1)	99 (22.3)	22 (5)	6 (1.4)
Mindfulness	12 (2.7)	67 (15.1)	210 (47.3)	116 (26.1)	37 (8.3)
Social connection	247 (55.6)	142 (32)	48 (10.8)	6 (1.4)	1 (0.2)

**Table 4:** Summary of participant’s program modality first preference (*N* = 444) [*N* (%)].

Variable	First preference
Storybook	77 (17.3)
Electronic application	56 (12.6)
Short film	63 (14.2)
Sensory activity	49 (11)
Arts-based delivery	52 (11.7)
Play-based delivery	31 (7)
Game-based delivery	47(10.6)
Music-based delivery	32 (7.2)
Excursion/activity-based delivery	34 (7.7)
Other	3 (0.7)

**Table 5:** Summary of participant’s agreeableness to potential features, topics and barriers to implementing a program (*N* = 444) [*N* (%)].

Variable	Strongly agree	Agree	Neutral	Disagree	Strongly disagree	N/A
Program features						
User-friendly materials	314 (70.7)	118 (26.6)	7 (1.6)	0 (0)	4 (0.9)	
Accessible	297 (66.9)	129 (29.1)	14 (3.2)	0 (0)	3 (0.7)	
Parent involvement	194 (43.7)	201 (45.3)	44 (9.9)	1 (0.2)	2 (0.5)	
Professional support	177 (39.9)	187 (42.1)	72 (16.2)	4 (0.9)	3 (0.7)	
Tailored content	181 (40.8)	175 (39.4)	80 (18)	3 (0.7)	4 (0.9)	
Affordability	308 (69.4)	111 (25)	22 (5)	0 (0)	3 (0.7)	
Program topics	274 (61.7)	154 (34.7)	13 (2.9)	0 (0)	2 (0.5)	
Lifestyle habits	274 (61.7)	154 (34.7)	13 (2.9)	0 (0)	2 (0.5)	
Social engagement	258 (58.1)	167 (37.6)	14 (3.2)	2 (0.5)	3 (0.7)	
Brain health	262 (59)	161 (36.3)	18 (4.1)	1 (0.2)	2 (0.5)	
Stress management	325 (73.2)	99 (22.3)	14 (3.2)	0 (0)	5 (1.1)	
Sleep hygiene	294 (66.2)	132 (29.7)	15 (3.4)	0 (0)	3 (0.7)	
Physical activity	275 (61.9)	150 (33.8)	15 (3.4)	0 (0)	3 (0.7)	
Barriers						
Time	66 (14.9)	142 (32)	143 (32.2)	75 (16.9)	17 (3.8)	1 (0.2)
Cost	84 (18.9)	163 (36.7)	99 (22.3)	79 (17.8)	18 (4.1)	1 (0.2)
Accessibility	196 (44.1)	222 (50)	20 (4.5)	1 (0.2)	3 (0.7)	1 (0.2)
Lack of knowledge	159 (35.8)	203 (45.7)	70 (15.8)	8 (1.8)	1 (0.2)	3 (0.7)
Lack of understanding	15 (3.4)	90 (20.3)	123 (27.7)	157 (35.4)	53 (11.9)	6 (1.4)
Unpredictable schedule	32 (7.3)	122 (27.5)	129 (29.1)	129 (29.1)	29 (6.5)	3 (0.7)
Lack of accountability	12 (2.7)	88 (19.8)	118 (26.6)	176 (39.6)	47 (10.6)	3 (0.7)
Not relevant	6 (1.4)	24 (5.4)	55 (12.4)	216 (48.6)	142 (32)	1 (0.2)
Lack of cultural considerations	39 (8.8)	102 (23)	143 (32.2)	91 (20.5)	38 (8.6)	31 (7)
Lack of confidence	8 (1.8)	71 (16)	86 (19.4)	210 (47.3)	69 (15.5)	0 (0)

**Table 6:** Multiple linear regression analysis with program uptake as criterion variable (*N* = 427).

Variable	*B*	SE *B*	*β*	95% CI for *B*		*p*	*sr*	Unique variance (%)
				LB	UB			
Constant	1.33	0.28		0.79	1.88	<0.001		
Parent gender	0.05	0.12	0.01	−0.19	0.28	0.701	0.01	0.0
Parent age	0.01	0.01	0.06	−0.01	0.02	0.155	0.05	0.3
Socioeconomic status	0.01	0.02	0.01	−0.03	0.04	0.720	0.01	0.0
Parent level of education	0.02	0.02	0.03	−0.03	0.06	0.410	0.03	0.1
Existing motivators of parent	0.08	0.06	0.05	−0.03	0.19	0.136	0.05	0.3
Number of perceived barriers	−0.04	0.02	−0.10	−0.07	−0.01	0.005	−0.10	1.0
Perceived usefulness for child	0.24	0.04	0.28	0.16	0.32	<0.001	0.20	4.0
Perceived usefulness for parent	0.38	0.04	0.45	0.28	0.46	<0.001	0.32	10.1
Child age	−0.01	0.01	−0.03	−0.03	0.02	0.512	−0.02	0.0

### 3.2 Multiple linear regression

Multiple linear regression showed that the overall model was statistically significant, *F*(9,417) = 44.57, *p* < 0.001, explaining large variance in intended uptake according to Cohen’s (35) benchmarks, *R*^2^ = 0.49, adjusted *R*^2^ = 0.48 (Table 6). Perceived usefulness of the program for parents was the most important predictor of program uptake (sr = 0.32), explaining 10.1% of the unique variance in program uptake. Additionally, the perceived usefulness of a program for children was also a significant predictor (sr = 0.20), explaining 4.0% unique variance in program uptake. Finally, the total number of perceived barriers was also a significant predictor (sr = −0.10), explaining 1.0% unique variance in intended uptake. The remaining independent variables were not significant predictors of intended program uptake.

### 3.3 Content analysis

**Figure 1:**
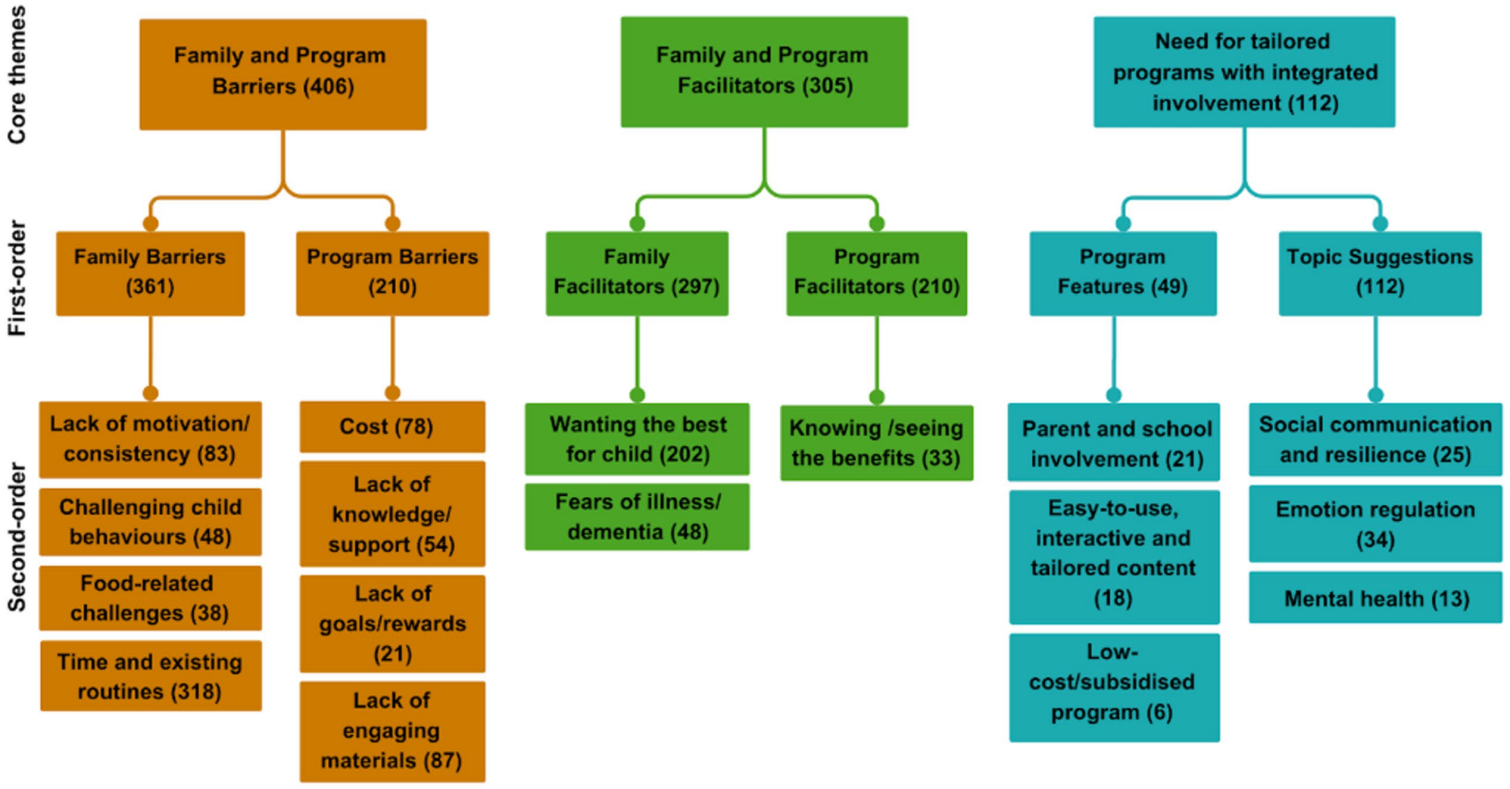
Core themes, first-order and second-order categories with number of responses per category. Information in brackets indicates the number of responses per category.

Three core themes were identified, with a total of six first-order categories and 17 second-order categories (Figure 1).

#### 3.3.1 Family and program barriers

Four hundred and six participants reported a range of barriers when asked about the short-and-long term implementation of a prospective program. These barriers consistently displayed a pattern of family-based and program-based barriers.

##### 3.3.1.1 Family barriers

A total of 361 participants shared insights into family-specific barriers. The most common barrier was a lack of time and existing routines (*n* = 318/361, 88.1%). Many participants described how out-of-school activities and responsibilities, like dinner and homework, made it difficult to imagine implementing a new program. Participant 30 stated *“Time - there’s so much between work and school and friends and activities and homework and all the things!.”* In addition to time and routine pressures, participants also noted concerns about their ability to implement certain topics (*n* = 38/361, 10.5%), particularly around food and eating habits, as Participant 26 and 84, respectively, noted *“I struggle with motivating them to make healthy food choices”* and “*My child loves junk food and is not willing to eat healthy.”* These comments point to a broader challenge related to children’s resistance or difficult behaviors when engaging with a program (*n* = 48/361, 13.3%), as stated by Participant 79 “*Refusal from the child*” and Participant 240 “*Child is not following instructions*.”

When thinking about sustaining a program beyond 3 months, participants highlighted concerns around maintaining motivation and consistency with the program and fostering healthy behaviors (*n* = 83/361, 23%). Participant 84 noted *“Motivation for the plan - if it is not engaging and becomes a struggle to implement, I will not finish it/force it.”* and Participant 337 stated *“Staying consistent even in school holidays, sickness, extra curriculars. It’s easy for routines to slip.”* Participant 342 further highlighted *“I suppose just falling back into old habits! Sticking with a change, even a good one, can be hard for me and for them.”*

##### 3.3.1.2 Program barriers

Program-specific barriers were identified by 210 participants. A frequently identified barrier was non-engaging and non-interactive materials (*n* = 87/210, 41.4%). As noted by Participant 55 “*The main challenge is if the program is not interactive enough to children”* and further stated by Participant 75 *“Engaging resources that keep the conversation alive.”* Alongside this, concerns around the cost of a program were flagged (*n* = 78/210, 34.1%), with Participant 202 explaining *“There is often a financial barrier at the moment that could hinder our access to active programs for our child.”* and Participant 18 *“Cost may be a barrier for entry for many families which may make this another program exclusively for people with ‘money’.”* Participants also noted the importance of a program prioritizing the sustainment of motivation through goals and rewards (*n* = 21/210, 10%). Participant 82 explained that *“They [children] will need to see/hear/understand the benefits and then be personally motivated to implement changes.”* Similarly, Participant 313 mentioned *“If they [children] find it boring, or cannot see the benefits.”* The need for parents to be informed about the program and have access to support during implementation was frequently mentioned (*n* = 54/210, 25.7%). Participant 375 expressed *“I do not think we [parents] are prepared with adequate resources and training to help our child.”* and Participant 339 *“Parents may need a lot of guidance and to be held accountable to continue the program with their children.”*

#### 3.3.2 Family and program facilitators

Three hundred and five participants reported existing motivators to making lifestyle changes for their children in addition to those a program can foster. Responses consistently displayed a pattern of family-based and program-centered facilitators.

##### 3.3.2.1 Family facilitators

Almost all participants noted family-based facilitators. Over half noted their child’s healthy ageing as an incentive to engage in a program (*n* = 202/305, 66.2%). Participants 231 and 61 explained this respectively, *“I want my children to have a beautiful, healthy life and not suffer from illnesses”* and *“I want to offer any and all options that will benefit a healthier lifestyle for my child.”* Similarly, parents raised concerns pertaining to risk of illness and neurodegenerative diseases (*n* = 48/305, 15.7%), whether that be from hereditary-based or general non-specific perspectives. Participant 3 noted *“Dementia runs in our family”* and Participant 54 stated non-specifically, *“Fear of dementia and Alzheimer’s later in life.”*

###### 3.3.2.2 Program facilitators

A minority of participants highlighted important components of a program that would increase their likelihood of engaging with the content. Some participants mentioned that seeing and believing the benefits of a program would motivate them to make healthy lifestyle changes (*n* = 33/305, 10.8%). Participant 31 noted *“Seeing the positive results from my children”* and Participant 278 “See the benefits.” To prioritize implementation, participants expressed the need for programs to be evidence-based with tangible outcomes. As participant 182 stated *“If the new habits make a difference and help them to be calmer, then I would keep at it”* and highlighted by Participant 183 *“simple stats that help remind us of why this is important would be motivating.”*

#### 3.3.3 Need for tailored programs with integrated involvement

One hundred and twelve participants suggested additional features for a program with a subset including additional topics.

##### 3.3.3.1 Program features

Forty-nine participants provided suggestions on program features to be considered when designing a program. Participants highlighted the need for tailored programs that connect both school learning to parent knowledge and involvement (*n* = 21/49, 42.9%). Participant 12 highlighted the importance of parents being key implementers for change, “*Definitely kids listen more to other people, but parents should know the information as well to implement the changes.”* Participant 42 further acknowledged that school is a useful resource when providing this important information, *“I think teachers and schools should also be involved in implementing some of the activities or strategies … as children spend a large portion of their days at school.”* While emphasizing the connectedness between home and school, another subset of participants expressed the importance of user-friendly and self-directed programs so that child(ren) can also independently engage with materials where necessary (*n* = 18/49, 36.7%). Participant 48 stated *“… If it is user friendly … then it will be used by more.,”* with Participant 5 further suggesting, *“A self-directed program that could be implemented….”* Other participants highlighted the need to support children’s ability to focus, making it an enjoyable learning experience, Participant 33 stated *“Keep it short … After a full day of school most kids are tired and not able to focus at length, especially if there is reading/writing involved.”* This gives rise to another common idea, the need for interactive content and materials, with participants suggesting hands-on approaches for engagement as a suitable approach. Participant 5 suggested *“Live demos”* while Participant 2 listed *“case-based, real-life examples.”*

###### 3.3.3.2 Topic suggestions

When thinking about interactive content, tailored content was seen as an important component of this, with participants noting further relevant topics for a program. Mental health was frequently suggested with participants expressing the importance of children having the skills and knowledge to support their mental health while also having an understanding of external support (*n* = 13/112, 11.6%). Participant 103 suggested *“Something about psychological wellbeing perhaps?,”* and Participant 88 noted *“How to identify when the brain is not happy and where to go for help.”* In addition to this, participants suggested focusing on children’s ability to communicate with peers confidently, noting the importance of healthy social dynamics and developing the skills of resilience (*n* = 25/112, 22.3%). Participant 92 suggesting *“Practicing healthy interactions between other peers and with parents …”* and Participant 60 explaining *“Resilience and healthy social interactions. Promoting respectful behaviors to reduce risk of bullying, exclusion behaviors.”* A topic related to increasing children’s ability to identify and manage emotions was frequently noted, highlighting the value of a topic that covered emotion regulation (*n* = 34/112, 30.4%). As described by Participant 59, *“Expressing emotions [and] utilizing easy to understand words for children”* and Participant 108, *“Understanding how to identify emotion to help identify [what] you need to regulate.”*

## 4 Discussion

The present study explored parental willingness to engage with a brain health program designed for primary school-aged children. It aimed to understand the barriers and facilitators influencing parental and child engagement, while exploring their preferences on the main components of an educational program. Findings revealed that parents were largely open and willing to participate in a prospective program despite a myriad of potential barriers. To facilitate adoption and manage these multifaceted barriers, findings suggested that a program needs to be tailored, interactive and comprehensive, prioritizing parental awareness and involvement, through a program connecting school-based learning into the home with parents.

Results showed that higher perceived usefulness, for both parents and children, and fewer perceived barriers were associated with higher willingness to participate in a program. Qualitative findings suggest that when parents believe a program will contribute positively to their children’s lifestyle habits, their likelihood of engaging with the program increases. Similarly, parents who viewed the materials as personally relevant and beneficial were more inclined to exhibit openness to adopt a program for their children. While not used to guide the selection of regression predictors, these dynamics resonate with the broader literature on health behavior and show conceptual alignment with constructs of the theory of planned behavior (29). Specifically, the functions of attitude and perceived behavioral control in shaping behavior may be evident, as parents positively evaluated the program’s relevance and potential impact, strengthening their intentions for implementation (29). Furthermore, the impact of perceived barriers emphasizes the important role of behavioral control in shaping parents’ confidence and capability to support future program uptake. These results align with prior research and underscore the importance of upskilling parents by increasing their knowledge about brain health and its relevance, while also addressing delivery and implementation challenges (3–5).

The most frequently reported barriers differed between the quantitative and qualitative findings, likely reflecting differences in measurement approach, with close-ended items capturing predefined barriers and open-ended responses offering more subjective constraints. As such, parents identified a constellation of barriers that spanned both family life and the structural conditions of program delivery. Consistent with earlier studies with preschool parents (3, 4), family-based barriers included entrenched household routines, children’s behavioral challenges, and parents’ limited knowledge or confidence in facilitating health-related change. Programmatic barriers included cost, limited accessibility, and a perceived lack of engaging, age-appropriate materials. These dual layers of constraint highlight the necessity of addressing both micro-level (family) and meso-level (programmatic and institutional) factors if an educational brain health program is to be effective. These findings are consistent with previous program implementation research where meso-and-macro level factors impacted upon the adoption and success of a physical activity program for school-aged children (38). These findings reveal the importance of an integrated strategy, starting from a macro-level (government/environment), that addresses these anticipated hurdles to ensure optimal accessibility and discernible impact on public health.

Taken together, findings highlight the need for an integrated, community-based program delivered across school and home settings, with active collaboration between parents, school and health professionals. Consistent with previous research, while parents recognized their role in encouraging health behaviors, they largely considered schools as primarily responsible for health-based education (30, 31). Our findings extend this perspective and reinforce Epstein et al.’s (32) framework of parental involvement, suggesting that well-informed, actively involved parents are more likely to scaffold school-based learning at home, thereby making them critical enablers to amplify program impact (33, 34). This joint effort of micro-and meso-level factors underscores the necessity of a program that connects school, family and professional support cohesively.

### 4.1 Limitations and future directions

The present study has several limitations for consideration. Notably, there was a significant unequal distribution of gender, with only 4.5% of participants identifying as male. While the uptake of female participants does indicate the role mothers play in caring for and engaging with children’s learning and engagement, this underrepresentation of fathers raises concerns about the imbalance of perspectives within the context of brain health and may limit the view of the broader family unit and generalizability of the findings. Future investigations would benefit from incorporating fathers’ and other caregivers’ views to provide broader perspectives on decision-making and responsibility within families, while enhancing generalizability of findings. The cross-sectional and self-report design of the study further limits the causality and generalizability of findings. While the intention was to obtain a snapshot of parents’ perspectives at a single point in time, it is important to note that interpretation of findings should be understood as associative and not causal. Further, the universal endorsement rate of brain health education (100%) warrants cautious interpretation as this finding may reflect ceiling effects or social desirability bias, particularly given the online survey format and positive framing and explanations of the topic. As such, the results may overestimate participants’ true levels of endorsement and limit the ability to detect variability in perspectives. Moreover, although incentives are commonly used in survey research to enhance participation, their use may have influenced responses and increased the risk of positive response bias from participants. Future research should focus on gathering richer qualitative data as while optional open-ended survey questions offered initial insights into parental perspectives longitudinal, ethnographic, or interview-based studies could provide deeper understandings of the lived realities shaping engagement with future brain health initiatives.

### 4.2 Strengths and implications

Despite these limitations, this study offers important contributions, it represents, to our knowledge, the first systematic exploration of dementia risk reduction initiatives oriented toward primary school-aged children, and aims to situate brain health in the context of early lifestyle formation. The large, geographically diverse sample enhances the robustness of the findings and provides a national snapshot of parental perspectives. These findings will not only promote discussion and further inquiry into this area but also have the potential to influence policy makers and institutions at large to prioritize and invest in the development of programs for this age group. These findings suggest that preventative health efforts should incorporate a cohesive brain health framework that intersects with parental aspirations, structural inequalities, and the everyday pressures of family time. Effective programs must therefore operate across settings (e.g., schools, homes, communities) while addressing both material barriers (e.g., cost, time accessibility) and symbolic concerns (e.g., perceived relevance, identity as a “good parent”), focusing on a broad range of health-related topics (e.g., physical activity, sleep hygiene, wellbeing).

### 4.3 Conclusion

The current study aimed to investigate parents’ willingness to engage with a prospective educational brain health program designed for primary school-aged children, while exploring their perceived barriers, facilitators and preferences on the main components of a program. Findings showed that parents clearly recognized the importance of preventive programs, even while anticipating significant structural and familial barriers to implementation. This illustrates both the promise and the complexity of embedding brain health education in childhood and underscores the importance of foregrounding parents’ voices in program design. These findings suggest the need for health and educational systems, alongside policymakers, to invest in programs that do not simply target children, but also strengthen the capacity of parents, families and communities to support long-term brain health and wellbeing. This study represents not only a scientifically grounded strategy, but also a timely policy imperative. It contributes to a growing body of research that calls for preventive strategies that are life-course-oriented and attentive to early dementia prevention.

## Data Availability

The datasets presented in this article are not readily available because per approved data management plan, the dataset cannot be shared publicly. Requests to access the datasets should be directed to Joyce Siette, Joyce.Siette@westernsydney.edu.au.
