# Supplementary material for: Parental perspectives on brain health education for primary school-aged children: a cross-sectional study
Source: Front Public Health. 2026 Jul 6;14:1829475. doi: 10.3389/fpubh.2026.1829475 (PMC13381779; doi:10.3389/fpubh.2026.1829475)
Supplement: Supplementary file 1 [file Supplementary_file_1.docx]

Supplementary Material

# Supplementary Data

Supplementary Material should be uploaded separately on submission. Please include any supplementary data, figures and/or tables.

Supplementary material is not typeset so please ensure that all information is clearly presented, the appropriate caption is included in the file and not in the manuscript, and that the style conforms to the rest of the article.

**Survey**

Start of Block: Introduction/General Information

Hello!
You are invited to participate in a research study to explore parents’ acceptability of brain health programs for primary school children. This project is being led by researchers from the MARCS Institute for Brain, Behaviour and Development at Western Sydney University.

NEXT PAGE

This 15-minute anonymous survey will seek your opinion and attitudes towards childhood brain health educational programs. This information will help us to develop useful educational health programs to support primary school aged children. Your participation is entirely voluntary. You can exit our online anonymous survey at any given time.

NEXT PAGE

Upon completion, you can enter a draw to win one of two $100 gift vouchers. You will receive access to our bespoke brain health activity pack designed for your child(ren). There are two different booklets. One is for Years K to 2 and the other for Years 3 to 6. Additionally, you will have access to a parent information sheet with more details about brain health and our current research for your interest.

NEXT PAGE

To participate in the study, you will need to be:

- Over 18 years old
- Able to communicate in English
- Able to provide consent
- A parent of a child currently attending primary school (usually aged 6-11 years old)

NEXT PAGE

It is anticipated that the overall results of this project will be published and/or presented in a variety of forums. Your personal details, such as your name or address, will not be asked, so that we can ensure your confidentiality.    Please be assured that only the researchers will have access to the raw data you provide. However, your data may be used in other related projects for up to five years.  We anticipate these projects to be similar in nature and to answer additional research questions that are yet to arise. Following this period, the data and information you have provided will be securely disposed of. You can find out more information about the study here: Information sheet If you have any questions, please contact Lily Montague (Master of Clinical Psychology Candidate): 20185190@student.westernsydney.edu.au

End of Block: Introduction/General Information

Start of Block: Consent

  I consent to be a part of this online survey  (1)

 I consent to provide my data as it was described to be used for this project  (2)

 I consent for my data and information provided to be used in this project and other related projects for an extended period of time  (3)

 I understand that my involvement is confidential and that the information gained during the study may be published and stored for other research use but no information about me will be used in any way that reveals my identity  (4)

 I understand that I can withdraw from the study at any time without affecting my relationship with the researcher/s, and any organisation involved, now or in the future  (5)

NEXT PAGE

I am over the age of 18 years old

 Yes  (1)

 No  (2)

NEXT PAGE

How many children do you have?

1  (1)

2  (2)

3  (3)

4  (4)

5 +  (5) __________________________________________________

NEXT PAGE

Are you a parent of a child, or children, currently attending primary school?

Yes  (1)

No  (2)

 NEXT PAGE

Display this question: If How many children do you have? = 1 SELECTED

How old is your child and what is their gender?

- Child 1 age and gender:  (1) ________________________________________________

NEXT PAGE

Display this question: If How many children do you have? = 2 SELECTED

 How old are each of your children and what are their genders?

- Child 1 age and gender:  (1) ________________________________________________
- Child 2 age and gender:  (2) ________________________________________________

NEXT PAGE

Display this question: If How many children do you have? = 3 SELECTED

How old are each of your children and what are their genders?

- Child 1 age and gender:  (1) _________________________________________________
- Child 2 age and gender:  (2) _________________________________________________
- Child 3 age and gender:  (3) ________________________________________________

NEXT PAGE

Display this question: If How many children do you have? = 4 SELECTED

How old are each of your children and what are their genders?

- Child 1 age and gender:  (1) ________________________________________________
- Child 2 age and gender:  (2) _________________________________________________
- Child 3 age and gender:  (3) _________________________________________________
- Child 4 age and gender:  (4) _________________________________________________

NEXT PAGE

Display this question: If How many children do you have? = 5 + SELECTED

How old are each of your children and what are their genders?

- Child 1 age and gender:  (1) _________________________________________________
- Child 2 age and gender:  (2) _________________________________________________
- Child 3 age and gender:  (3) _________________________________________________
- Child 4 age and gender:  (4) _________________________________________________
- Child 5 age and gender:  (5) _________________________________________________
- Child 6 + age and gender:  (6) _______________________________________________

NEXT PAGE

How many children do you have that currently attend primary school?

- 0  (7)
- 1  (1)
- 2  (2)
- 3  (3)
- 4  (4)
- 5 +  (5)

End of Block: Consent/Eligibility

Start of Block: Demographics

What is a brain health program?   
Brain health programs aim to support healthy ageing by promoting education and positive lifestyle changes. They have typically targeted older age groups. Some programs you may have heard of include Brain Balance, LearningRx, and MindUP. Our research focuses on developing educational primary school programs for children aged between 6 and 11 years to help build holistic healthy lifestyle habits at an earlier age. This will enable children to have stronger brains and mindsets on ageing well. We’re keen to know your opinion on how these programs can be developed and used at home and at school. First, a few questions about you.

NEXT PAGE

How old are you? ___________________________________________________

NEXT PAGE

 What do you identify as?

- Male  (1)
- Female  (2)
- Non-binary / third gender  (3)
- Prefer not to say  (4)

NEXT PAGE

What is your place of birth?

- Australia  (1)
- New Zealand  (2)
- United Kingdom  (3)
- Italy  (4)
- China  (5)
- Lebanon  (6)
- India  (7)
- Greece  (8)
- Other, please specify:  (9) __________________________________________________

NEXT PAGE

What is your postcode? __________________________________________________________

NEXT PAGE

What is the highest degree or level of education you have completed?

- Less than Year 12 or equivalent  (1)
- Year 12 or equivalent  (2)
- Vocational qualification  (3)
- Bachelor's degree  (4)
- Master's degree  (5)
- PhD or higher  (6)
- Prefer not to say  (7)

 NEXT PAGE

What is your employment status?

- Employed full time  (1)
- Employed part time  (2)
- Employed casual  (3)
- Unemployed looking for work  (4)
- Unemployed not looking for work  (5)
- Retired  (6)
- Student  (7)
- Disabled  (8)
- Prefer not to say  (9)

 NEXT PAGE

What structure below best explains your family?

- Married  (1)
- Single parent  (2)
- Defacto  (3)
- Other  (4) __________________________________________________

NEXT PAGE:

On average, how many hours per day do you spend with your child(ren) meaningfully?

- Less than 1 hour  (1)
- 1 to 3 hours  (2)
- 3 to 5 hours  (3)
- 5 + hours  (4)
- Other  (5) __________________________________________________

 NEXT PAGE

Has your child(ren) received or been exposed to information on brain health through any of these sources?  Please select all that applies.

- Preschool/Primary school education  (1)
- Community health programs (e.g., at the library, playgroups)  (2)
- Pediatrician or healthcare provider  (3)
- Online resources (e.g., from the Department of Education)  (4)
- Children's books  (5)
- Public health campaigns (e.g., at shopping centres)  (6)
- Educational apps and games  (7)
- Other  (8) __________________________________________________
- None  (9)

NEXT PAGE

Have you ever had a relative or someone close to you experience a brain-related disease or condition? (e.g., dementia, stroke, traumatic brain injury)

- Yes  (1)
- No  (2)
- Prefer not to say  (3)

 NEXT PAGE

Does/is your child(ren) aged 6 to 11 years likely to have a neurodiverse condition?

- Yes  (1)
- No  (2)
- Prefer not to say  (3)

NEXT PAGE

Display this question:,If How many children do you have that currently attend primary school? = 1 SELECTED

Below are some common habits that support brain development in children aged 6 to 11 years.   Please select whether your child is currently engaged in these activities/practices.

|  | Never (1) | Rarely (2) | Sometimes (5) | Often (6) | Always (3) | N/A (4) |
| --- | --- | --- | --- | --- | --- | --- |
| My child(ren) engage(s) in appropriate sleep hygiene (e.g., consistent sleep schedule, limited screen time before bed, and sleep-conductive environment such as a dark, cool room) (1) | o | o | o | o | o | o |
| My child(ren) engage(s) in moderate physical activity (At least 1 hour per day) (2) | o | o | o | o | o | o |
| My child/ren engage(s) in healthy eating (A balanced diet with limited processed and sugary foods)  (3) | o | o | o | o | o | o |
| My child(ren) uses mindfulness and/or stress management behaviours (e.g., deep breathing, muscle relaxation and/or meditation) (5) | o | o | o | o | o | o |
| My child(ren) is/are sociable and enjoy engaging with peers both inside and outside of school (6) | o | o | o | o | o | o |

What is the age of the child you completed this table for?

- 6  (1)
- 7  (2)
- 8  (3)
- 9  (4)
- 10  (5)
- 11  (6)

NEXT PAGE

Display this question: If How many children do you have that currently attend primary school? = 2 SELECTED

Below are some common habits that support brain development in children aged 6 to 11 years. Please select whether your child is currently engaged in these activities/practices.  Please complete the separate set of these questions below for each child.

|  | Never (1) | Rarely (2) | Sometimes (5) | Often (6) | Always (3) | N/A (4) |
| --- | --- | --- | --- | --- | --- | --- |
| My child(ren) engage(s) in appropriate sleep hygiene (e.g., consistent sleep schedule, limited screen time before bed, and sleep-conductive environment such as a dark, cool room) (1) | o | o | o | o | o | o |
| My child(ren) engage(s) in moderate physical activity (At least 1 hour per day) (2) | o | o | o | o | o | o |
| My child/ren engage(s) in healthy eating (A balanced diet with limited processed and sugary foods)  (3) | o | o | o | o | o | o |
| My child(ren) uses mindfulness and/or stress management behaviours (e.g., deep breathing, muscle relaxation and/or meditation) (5) | o | o | o | o | o | o |
| My child(ren) is/are sociable and enjoy engaging with peers both inside and outside of school (6) | o | o | o | o | o | o |

What is the age of the child you completed this table for?

- 6  (1)
- 7  (2)
- 8  (3)
- 9  (4)
- 10  (5)
- 11  (6)

NEXT PAGE

Display this question: If How many children do you have that currently attend primary school? = 2 SELECTED

Below are some common habits that support brain development in children aged 6 to 11 years. Please select whether your child is currently engaged in these activities/practices.  Please complete the separate set of these questions below for each child.

|  | Never (1) | Rarely (2) | Sometimes (5) | Often (6) | Always (3) | N/A (4) |
| --- | --- | --- | --- | --- | --- | --- |
| My child(ren) engage(s) in appropriate sleep hygiene (e.g., consistent sleep schedule, limited screen time before bed, and sleep-conductive environment such as a dark, cool room) (1) | o | o | o | o | o | o |
| My child(ren) engage(s) in moderate physical activity (At least 1 hour per day) (2) | o | o | o | o | o | o |
| My child/ren engage(s) in healthy eating (A balanced diet with limited processed and sugary foods)  (3) | o | o | o | o | o | o |
| My child(ren) uses mindfulness and/or stress management behaviours (e.g., deep breathing, muscle relaxation and/or meditation) (5) | o | o | o | o | o | o |
| My child(ren) is/are sociable and enjoy engaging with peers both inside and outside of school (6) | o | o | o | o | o | o |

What is the age of the child you completed this table for?

- 6  (1)
- 7  (2)
- 8  (3)
- 9  (4)
- 10  (5)
- 11  (6)

 NEXT PAGE

Display this question: If How many children do you have that currently attend primary school? = 3 SELECTED

Below are some common habits that support brain development in children aged 6 to 11 years. Please select whether your child is currently engaged in these activities/practices.   Please complete the separate set of these questions below for each child.

|  | Never (1) | Rarely (2) | Sometimes (5) | Often (6) | Always (3) | N/A (4) |
| --- | --- | --- | --- | --- | --- | --- |
| My child(ren) engage(s) in appropriate sleep hygiene (e.g., consistent sleep schedule, limited screen time before bed, and sleep-conductive environment such as a dark, cool room) (1) | o | o | o | o | o | o |
| My child(ren) engage(s) in moderate physical activity (At least 1 hour per day) (2) | o | o | o | o | o | o |
| My child/ren engage(s) in healthy eating (A balanced diet with limited processed and sugary foods)  (3) | o | o | o | o | o | o |
| My child(ren) uses mindfulness and/or stress management behaviours (e.g., deep breathing, muscle relaxation and/or meditation) (5) | o | o | o | o | o | o |
| My child(ren) is/are sociable and enjoy engaging with peers both inside and outside of school (6) | o | o | o | o | o | o |

What is the age of the child you completed this table for?

 6  (1)

7  (2)

 8  (3)

9  (4)

10  (5)

11  (6)

NEXT PAGE

 Display this question:If How many children do you have that currently attend primary school? = 3 SELECTED

Below are some common habits that support brain development in children aged 6 to 11 years. Please select whether your child is currently engaged in these activities/practices.   Please complete the separate set of these questions below for each child.

|  | Never (1) | Rarely (2) | Sometimes (5) | Often (6) | Always (3) | N/A (4) |
| --- | --- | --- | --- | --- | --- | --- |
| My child(ren) engage(s) in appropriate sleep hygiene (e.g., consistent sleep schedule, limited screen time before bed, and sleep-conductive environment such as a dark, cool room) (1) | o | o | o | o | o | o |
| My child(ren) engage(s) in moderate physical activity (At least 1 hour per day) (2) | o | o | o | o | o | o |
| My child/ren engage(s) in healthy eating (A balanced diet with limited processed and sugary foods)  (3) | o | o | o | o | o | o |
| My child(ren) uses mindfulness and/or stress management behaviours (e.g., deep breathing, muscle relaxation and/or meditation) (5) | o | o | o | o | o | o |
| My child(ren) is/are sociable and enjoy engaging with peers both inside and outside of school (6) | o | o | o | o | o | o |

What is the age of the child you completed this table for?

- 6  (1)
- 7  (2)
- 8  (3)
- 9  (4)
- 10  (5)
- 11  (6)

 NEXT PAGE

Display this question: If How many children do you have that currently attend primary school? = 3 SELECTED

Below are some common habits that support brain development in children aged 6 to 11 years. Please select whether your child is currently engaged in these activities/practices.   Please complete the separate set of these questions below for each child.

|  | Never (1) | Rarely (2) | Sometimes (5) | Often (6) | Always (3) | N/A (4) |
| --- | --- | --- | --- | --- | --- | --- |
| My child(ren) engage(s) in appropriate sleep hygiene (e.g., consistent sleep schedule, limited screen time before bed, and sleep-conductive environment such as a dark, cool room) (1) | o | o | o | o | o | o |
| My child(ren) engage(s) in moderate physical activity (At least 1 hour per day) (2) | o | o | o | o | o | o |
| My child/ren engage(s) in healthy eating (A balanced diet with limited processed and sugary foods)  (3) | o | o | o | o | o | o |
| My child(ren) uses mindfulness and/or stress management behaviours (e.g., deep breathing, muscle relaxation and/or meditation) (5) | o | o | o | o | o | o |
| My child(ren) is/are sociable and enjoy engaging with peers both inside and outside of school (6) | o | o | o | o | o | o |

What is the age of the child you completed this table for?

- 6  (1)
- 7  (2)
- 8  (3)
- 9  (4)
- 10  (5)
- 11  (6)

NEXT PAGE

Display this question: If How many children do you have that currently attend primary school? = 4 SELECTED

Below are some common habits that support brain development in children aged 6 to 11 years. Please select whether your child is currently engaged in these activities/practices.   Please complete the separate set of these questions below for each child.

|  | Never (1) | Rarely (2) | Sometimes (5) | Often (6) | Always (3) | N/A (4) |
| --- | --- | --- | --- | --- | --- | --- |
| My child(ren) engage(s) in appropriate sleep hygiene (e.g., consistent sleep schedule, limited screen time before bed, and sleep-conductive environment such as a dark, cool room) (1) | o | o | o | o | o | o |
| My child(ren) engage(s) in moderate physical activity (At least 1 hour per day) (2) | o | o | o | o | o | o |
| My child/ren engage(s) in healthy eating (A balanced diet with limited processed and sugary foods)  (3) | o | o | o | o | o | o |
| My child(ren) uses mindfulness and/or stress management behaviours (e.g., deep breathing, muscle relaxation and/or meditation) (5) | o | o | o | o | o | o |
| My child(ren) is/are sociable and enjoy engaging with peers both inside and outside of school (6) | o | o | o | o | o | o |

What is the age of the child you completed this table for?

- 6  (1)
- 7  (2)
- 8  (3)
- 9  (4)
- 10  (5)
- 11  (6)

NEXT PAGE

Display this question: If How many children do you have that currently attend primary school? = 4 SELECTED

Below are some common habits that support brain development in children aged 6 to 11 years. Please select whether your child is currently engaged in these activities/practices.   Please complete the separate set of these questions below for each child.

|  | Never (1) | Rarely (2) | Sometimes (5) | Often (6) | Always (3) | N/A (4) |
| --- | --- | --- | --- | --- | --- | --- |
| My child(ren) engage(s) in appropriate sleep hygiene (e.g., consistent sleep schedule, limited screen time before bed, and sleep-conductive environment such as a dark, cool room) (1) | o | o | o | o | o | o |
| My child(ren) engage(s) in moderate physical activity (At least 1 hour per day) (2) | o | o | o | o | o | o |
| My child/ren engage(s) in healthy eating (A balanced diet with limited processed and sugary foods)  (3) | o | o | o | o | o | o |
| My child(ren) uses mindfulness and/or stress management behaviours (e.g., deep breathing, muscle relaxation and/or meditation) (5) | o | o | o | o | o | o |
| My child(ren) is/are sociable and enjoy engaging with peers both inside and outside of school (6) | o | o | o | o | o | o |

What is the age of the child you completed this table for?

- 6  (1)
- 7  (2)
- 8  (3)
- 9  (4)
- 10  (5)
- 11  (6)

NEXT PAGE

Display this question:If How many children do you have that currently attend primary school? = 4 SELECTED

Below are some common habits that support brain development in children aged 6 to 11 years. Please select whether your child is currently engaged in these activities/practices.   Please complete the separate set of these questions below for each child.

|  | Never (1) | Rarely (2) | Sometimes (5) | Often (6) | Always (3) | N/A (4) |
| --- | --- | --- | --- | --- | --- | --- |
| My child(ren) engage(s) in appropriate sleep hygiene (e.g., consistent sleep schedule, limited screen time before bed, and sleep-conductive environment such as a dark, cool room) (1) | o | o | o | o | o | o |
| My child(ren) engage(s) in moderate physical activity (At least 1 hour per day) (2) | o | o | o | o | o | o |
| My child/ren engage(s) in healthy eating (A balanced diet with limited processed and sugary foods)  (3) | o | o | o | o | o | o |
| My child(ren) uses mindfulness and/or stress management behaviours (e.g., deep breathing, muscle relaxation and/or meditation) (5) | o | o | o | o | o | o |
| My child(ren) is/are sociable and enjoy engaging with peers both inside and outside of school (6) | o | o | o | o | o | o |

What is the age of the child you completed this table for?

- 6  (1)
- 7  (2)
- 8  (3)
- 9  (4)
- 10  (5)
- 11  (6)

 NEXT PAGE

Display this question: If How many children do you have that currently attend primary school? = 4 SELECTED

Below are some common habits that support brain development in children aged 6 to 11 years. Please select whether your child is currently engaged in these activities/practices.   Please complete the separate set of these questions below for each child.

|  | Never (1) | Rarely (2) | Sometimes (5) | Often (6) | Always (3) | N/A (4) |
| --- | --- | --- | --- | --- | --- | --- |
| My child(ren) engage(s) in appropriate sleep hygiene (e.g., consistent sleep schedule, limited screen time before bed, and sleep-conductive environment such as a dark, cool room) (1) | o | o | o | o | o | o |
| My child(ren) engage(s) in moderate physical activity (At least 1 hour per day) (2) | o | o | o | o | o | o |
| My child/ren engage(s) in healthy eating (A balanced diet with limited processed and sugary foods)  (3) | o | o | o | o | o | o |
| My child(ren) uses mindfulness and/or stress management behaviours (e.g., deep breathing, muscle relaxation and/or meditation) (5) | o | o | o | o | o | o |
| My child(ren) is/are sociable and enjoy engaging with peers both inside and outside of school (6) | o | o | o | o | o | o |

What is the age of the child you completed this table for?

- 6  (1)
- 7  (2)
- 8  (3)
- 9  (4)
- 10  (5)
- 11  (6)

NEXT PAGE

Display this question: If How many children do you have that currently attend primary school? = 5 + SELECTED

Below are some common habits that support brain development in children aged 6 to 11 years. Please select whether your child is currently engaged in these activities/practices.   Please complete the separate set of these questions below for each child.

|  | Never (1) | Rarely (2) | Sometimes (5) | Often (6) | Always (3) | N/A (4) |
| --- | --- | --- | --- | --- | --- | --- |
| My child(ren) engage(s) in appropriate sleep hygiene (e.g., consistent sleep schedule, limited screen time before bed, and sleep-conductive environment such as a dark, cool room) (1) | o | o | o | o | o | o |
| My child(ren) engage(s) in moderate physical activity (At least 1 hour per day) (2) | o | o | o | o | o | o |
| My child/ren engage(s) in healthy eating (A balanced diet with limited processed and sugary foods)  (3) | o | o | o | o | o | o |
| My child(ren) uses mindfulness and/or stress management behaviours (e.g., deep breathing, muscle relaxation and/or meditation) (5) | o | o | o | o | o | o |
| My child(ren) is/are sociable and enjoy engaging with peers both inside and outside of school (6) | o | o | o | o | o | o |

What is the age of the child you completed this table for?

- 6  (1)
- 7  (2)
- 8  (3)
- 9  (4)
- 10  (5)
- 11  (6)

NEXT PAGE

Display this question: If How many children do you have that currently attend primary school? = 5 + SELECTED

Below are some common habits that support brain development in children aged 6 to 11 years. Please select whether your child is currently engaged in these activities/practices.   Please complete the separate set of these questions below for each child.

|  | Never (1) | Rarely (2) | Sometimes (5) | Often (6) | Always (3) | N/A (4) |
| --- | --- | --- | --- | --- | --- | --- |
| My child(ren) engage(s) in appropriate sleep hygiene (e.g., consistent sleep schedule, limited screen time before bed, and sleep-conductive environment such as a dark, cool room) (1) | o | o | o | o | o | o |
| My child(ren) engage(s) in moderate physical activity (At least 1 hour per day) (2) | o | o | o | o | o | o |
| My child/ren engage(s) in healthy eating (A balanced diet with limited processed and sugary foods)  (3) | o | o | o | o | o | o |
| My child(ren) uses mindfulness and/or stress management behaviours (e.g., deep breathing, muscle relaxation and/or meditation) (5) | o | o | o | o | o | o |
| My child(ren) is/are sociable and enjoy engaging with peers both inside and outside of school (6) | o | o | o | o | o | o |

What is the age of the child you completed this table for?

- 6  (1)
- 7  (2)
- 8  (3)
- 9  (4)
- 10  (5)
- 11  (6)

NEXT PAGE

Display this question: If How many children do you have that currently attend primary school? = 5 + SELECTED

Below are some common habits that support brain development in children aged 6 to 11 years. Please select whether your child is currently engaged in these activities/practices. Please complete the separate set of these questions below for each child.

|  | Never (1) | Rarely (2) | Sometimes (5) | Often (6) | Always (3) | N/A (4) |
| --- | --- | --- | --- | --- | --- | --- |
| My child(ren) engage(s) in appropriate sleep hygiene (e.g., consistent sleep schedule, limited screen time before bed, and sleep-conductive environment such as a dark, cool room) (1) | o | o | o | o | o | o |
| My child(ren) engage(s) in moderate physical activity (At least 1 hour per day) (2) | o | o | o | o | o | o |
| My child/ren engage(s) in healthy eating (A balanced diet with limited processed and sugary foods)  (3) | o | o | o | o | o | o |
| My child(ren) uses mindfulness and/or stress management behaviours (e.g., deep breathing, muscle relaxation and/or meditation) (5) | o | o | o | o | o | o |
| My child(ren) is/are sociable and enjoy engaging with peers both inside and outside of school (6) | o | o | o | o | o | o |

What is the age of the child you completed this table for?

- 6  (1)
- 7  (2)
- 8  (3)
- 9  (4)
- 10  (5)
- 11  (6)

NEXT PAGE

Display this question: If How many children do you have that currently attend primary school? = 5 + SELECTED

Below are some common habits that support brain development in children aged 6 to 11 years. Please select whether your child is currently engaged in these activities/practices.   Please complete the separate set of these questions below for each child.

|  | Never (1) | Rarely (2) | Sometimes (5) | Often (6) | Always (3) | N/A (4) |
| --- | --- | --- | --- | --- | --- | --- |
| My child(ren) engage(s) in appropriate sleep hygiene (e.g., consistent sleep schedule, limited screen time before bed, and sleep-conductive environment such as a dark, cool room) (1) | o | o | o | o | o | o |
| My child(ren) engage(s) in moderate physical activity (At least 1 hour per day) (2) | o | o | o | o | o | o |
| My child/ren engage(s) in healthy eating (A balanced diet with limited processed and sugary foods)  (3) | o | o | o | o | o | o |
| My child(ren) uses mindfulness and/or stress management behaviours (e.g., deep breathing, muscle relaxation and/or meditation) (5) | o | o | o | o | o | o |
| My child(ren) is/are sociable and enjoy engaging with peers both inside and outside of school (6) | o | o | o | o | o | o |

What is the age of the child you completed this table for?

- 6  (1)
- 7  (2)
- 8  (3)
- 9  (4)
- 10  (5)
- 11  (6)

NEXT PAGE

Display this question: If How many children do you have that currently attend primary school? = 5 + SELECTED

Below are some common habits that support brain development in children aged 6 to 11 years. Please select whether your child is currently engaged in these activities/practices.   Please complete the separate set of these questions below for each child.

|  | Never (1) | Rarely (2) | Sometimes (5) | Often (6) | Always (3) | N/A (4) |
| --- | --- | --- | --- | --- | --- | --- |
| My child(ren) engage(s) in appropriate sleep hygiene (e.g., consistent sleep schedule, limited screen time before bed, and sleep-conductive environment such as a dark, cool room) (1) | o | o | o | o | o | o |
| My child(ren) engage(s) in moderate physical activity (At least 1 hour per day) (2) | o | o | o | o | o | o |
| My child/ren engage(s) in healthy eating (A balanced diet with limited processed and sugary foods)  (3) | o | o | o | o | o | o |
| My child(ren) uses mindfulness and/or stress management behaviours (e.g., deep breathing, muscle relaxation and/or meditation) (5) | o | o | o | o | o | o |
| My child(ren) is/are sociable and enjoy engaging with peers both inside and outside of school (6) | o | o | o | o | o | o |

What is the age of the child you completed this table for?

- 6  (1)
- 7  (2)
- 8  (3)
- 9  (4)
- 10  (5)
- 11  (6)

NEXT PAGE

Display this question: If How many children do you have that currently attend primary school? = 5 + SELECTED

Below are some common habits that support brain development in children aged 6 to 11 years. Please select whether your child is currently engaged in these activities/practices.   Please complete the separate set of these questions below for each child.

|  | Never (1) | Rarely (2) | Sometimes (5) | Often (6) | Always (3) | N/A (4) |
| --- | --- | --- | --- | --- | --- | --- |
| My child(ren) engage(s) in appropriate sleep hygiene (e.g., consistent sleep schedule, limited screen time before bed, and sleep-conductive environment such as a dark, cool room) (1) | o | o | o | o | o | o |
| My child(ren) engage(s) in moderate physical activity (At least 1 hour per day) (2) | o | o | o | o | o | o |
| My child/ren engage(s) in healthy eating (A balanced diet with limited processed and sugary foods)  (3) | o | o | o | o | o | o |
| My child(ren) uses mindfulness and/or stress management behaviours (e.g., deep breathing, muscle relaxation and/or meditation) (5) | o | o | o | o | o | o |
| My child(ren) is/are sociable and enjoy engaging with peers both inside and outside of school (6) | o | o | o | o | o | o |

What is the age of the child you completed this table for?

- 6  (1)
- 7  (2)
- 8  (3)
- 9  (4)
- 10  (5)
- 11  (6)

End of Block: Demographics

Start of Block: Acceptability of BHP

You are over halfway done!
This part now asks you to provide your thoughts and opinions on brain health programs for primary school aged children. These programs aim to educate children and their families about the importance of healthy brains. It focuses on how lifestyle habits can build stronger brains and encourages children to engage in brain healthy behaviours.

NEXT PAGE

How useful do you think a program would be for your primary school aged child(ren)?

- Not useful at all  (1)
- Slightly useful  (2)
- Moderately useful  (3)
- Very useful  (4)
- Extremely useful  (5)

NEXT PAGE

How useful do you think a program would be for you as a parent?

- Not useful at all  (1)
- Slightly useful  (2)
- Moderately useful  (3)
- Very useful  (4)
- Extremely useful  (5)

NEXT PAGE

In the case a program is made, how likely are you to use a brain health program for your primary school aged child?

- Extremely unlikely  (1)
- Unlikely  (2)
- Neutral  (3)
- Likely  (4)
- Extremely likely  (5)

End of Block: Acceptability of BHP

Start of Block: Modalities and location/setting

The following questions ask for your thoughts and opinions on different delivery formats and locations where a brain health program for children could be offered. As a reminder: These programs aim to educate children and their families about the importance of healthy brains. It focuses on how lifestyle habits can build stronger brains, and encourages children to engage in brain healthy behaviours.

NEXT PAGE

Where would you prefer a brain health program to be held?  Note: you can select more than one answer

- Primary school  (1)
- Home  (2)
- Before and/or after school care  (3)
- Holiday program  (4)
- Other, please specify: (5) __________________________________________________

Could you please let us know why you prefer to have the program held in these spaces and/or locations?

__________________________________________________________________________________________________________________________________________________________________________________________________________________________________________

NEXT PAGE

Please rank the below potential formats of a program by dragging them in your order of preference.  (Note: 1 = most preferred, 10 = least preferred)

- ______ Story Book (1)
- ______ Electronic Application (2)
- ______ Short Animation Film (3)
- ______ Sensory Activity (4)
- ______ Arts Based Delivery (5)
- ______ Play Based Delivery (6)
- ______ Game Based Delivery (7)
- ______ Music Based Delivery (8)
- ______ Excursion/Activity Based (9)
- ______ Other (10)

Can you please explain why you ranked the above formats in that order?

__________________________________________________________________________________________________________________________________________________________________________________________________________________________________________

Do you have any other suggested formats that were not listed?

__________________________________________________________________________________________________________________________________________________________________________________________________________________________________________

End of Block: Part 4 - Modalities and location/setting

Start of Block: Part 5 - Program content

Almost finished!
Next, we would like to ask about the importance of various topics and features that could be included in a brain health program. As a reminder: These programs aim to educate children and their families about the importance of healthy brains. It focuses on how lifestyle habits can build stronger brains and encourages children to engage in brain healthy behaviours.

NEXT PAGE

Here are some topics that can be used in these programs. Please rate whether you think they should be included in brain health programs:

|  | Strongly Disagree (1) | Disagree (2) | Neutral (3) | Agree (4) | Strongly Agree (5) | N/A (6) |
| --- | --- | --- | --- | --- | --- | --- |
| General healthy lifestyle habits (1) | o | o | o | o | o | o |
| Social engagement (e.g., importance and encouraging interaction with friends/peers) (2) | o | o | o | o | o | o |
| Education (e.g., importance of learning and brain health for healthy ageing) (3) | o | o | o | o | o | o |
| Stress management (e.g., strategies like mindfulness and meditation to regulate emotions) (4) | o | o | o | o | o | o |
| Sleep hygiene (e.g., routine before bed, screen time limit, improving quality of sleep) (5) | o | o | o | o | o | o |
| Exercise (e.g., regular physical activity at least 1 hour per day) (6) | o | o | o | o | o | o |
| Healthy foods (e.g., balanced diet with limited processed and sugary foods) (7) | o | o | o | o | o | o |

Do you have any other suggestions for topics to be considered?

__________________________________________________________________________________________________________________________________________________________________________________________________________________________________________

NEXT PAGE

We would like to hear your thoughts on other features of this program, and how we can develop one that supports you and your children. Please rate how important the below brain health program features are to you:

|  | Strongly Disagree (1) | Disagree (2) | Neutral (3) | Agree (4) | Strongly Agree (5) | N/A (6) |
| --- | --- | --- | --- | --- | --- | --- |
| User friendly materials (e.g., engaging, easy to use and interactive)  (1) | o | o | o | o | o | o |
| Accessibility (e.g., convenient for family to engage in) (2) | o | o | o | o | o | o |
| Parent involvement (e.g., parents are aware of brain health programs positive outcomes and are involved in aspects of the program) (3) | o | o | o | o | o | o |
| Guidance from health care professionals (e.g., support and guidance from health professionals in using the programs) (4) | o | o | o | o | o | o |
| Tailored programs (e.g., tailored to your child’s needs and interests) (5) | o | o | o | o | o | o |
| Affordability (e.g., low-cost programs and materials)  (6) | o | o | o | o | o | o |

Do you have any suggestions on other program features, or would like to elaborate on any of the listed suggestions?

__________________________________________________________________________________________________________________________________________________________________________________________________________________________________________

End of Block: Part 5 - Program content

Start of Block: Part 6 - Barriers and Facilitators

This is the final part! This section asks you about any barriers you might experience when implementing a brain health program for child/ren. As a reminder: These programs aim to educate children and their families about the importance of healthy brains. It focuses on how lifestyle habits can build stronger brains and encourages children to engage in brain healthy behaviours.

NEXT PAGE

Brain health programs aim to support healthy habits like eating well, being active, and having a consistent sleep schedule. What barriers, if any, do you think might make it difficult to implement these changes at home with your child(ren)?

__________________________________________________________________________________________________________________________________________________________________________________________________________________________________________

NEXT PAGE

Here are some barriers that might stop you from implementing brain health programs with your child(ren). Please rate how much you agree or disagree with these statements.

|  | Strongly disagree (1) | Disagree (2) | Neutral (3) | Agree (4) | Strongly agree (5) | N/A (6) |
| --- | --- | --- | --- | --- | --- | --- |
| I don’t have time to implement a program at home. (1) | o | o | o | o | o | o |
| I do not feel confident in my ability to make lifestyle changes with my children. (2) | o | o | o | o | o | o |
| I don’t want to spend too much money on a brain health program. (3) | o | o | o | o | o | o |
| I want an easily accessible brain health program. (4) | o | o | o | o | o | o |
| I want to ensure I am educated on what to do with a brain health program, and its outcomes before I implement it. (5) | o | o | o | o | o | o |
| I do not quite understand what a brain health program is. (6) | o | o | o | o | o | o |
| I have an unpredictable schedule which will impact making lifestyle changes with my children. (7) | o | o | o | o | o | o |
| If no one is holding me accountable, I will not implement brain health programs in my home. (8) | o | o | o | o | o | o |
| Brain health is not something I worry about/want my child to be educated on. (9) | o | o | o | o | o | o |
| My family needs a program that aligns with and considers our cultural values and beliefs. (1\0) | o | o | o | o | o | o |

NEXT PAGE

What do you think are the main challenges that might prevent you, or your child(ren), from staying engaged in healthy lifestyle changes over the long term (3+ months)?

__________________________________________________________________________________________________________________________________________________________________________________________________________________________________________

NEXT PAGE

Are there any specific motivators that inspire you to make healthy lifestyle changes at home for your child(ren)?

- Yes  (1)
- No  (2)

NEXT PAGE

Display this question: If Are there any specific motivators that inspire you to make healthy lifestyle changes at home for... = Yes SELECTED

Could you tell us what motivates you to support healthy lifestyle habits for your child(ren)?

__________________________________________________________________________________________________________________________________________________________________________________________________________________________________________

NEXT PAGE

 Display this question: If Are there any specific motivators that inspire you to make healthy lifestyle changes at home for... = No SELECTED

Are there any reasons why you might feel hesitant, or less motivated, to support healthy lifestyle habits for your child(ren)?

__________________________________________________________________________________________________________________________________________________________________________________________________________________________________________

NEXT PAGE

Do you have any additional comments/feedback you would like to share with us?

__________________________________________________________________________________________________________________________________________________________________________________________________________________________________________

NEXT PAGE

End of Block: Part 6 - Barriers and Facilitators
